# Tree of Sex: A database of sexual systems

**DOI:** 10.1038/sdata.2014.15

**Published:** 2014-06-24

**Authors:** The Tree of Sex Consortium, Tia-Lynn Ashman, Doris Bachtrog, Heath Blackmon, Emma E Goldberg, Matthew W Hahn, Mark Kirkpatrick, Jun Kitano, Judith E Mank, Itay Mayrose, Ray Ming, Sarah P Otto, Catherine L Peichel, Matthew W Pennell, Nicolas Perrin, Laura Ross, Nicole Valenzuela, Jana C Vamosi

**Affiliations:** 1 Department Biological Sciences, University of Pittsburgh, Pittsburgh, PA 15260, USA; 2 Department of Integrative Biology, University of California, Berkeley, CA 94720, USA; 3 Department of Biology, University of Texas, Arlington, TX 76019, USA; 4 Department of Ecology, Evolution, and Behavior, University of Minnesota, Saint Paul, MN 55108, USA; 5 Department of Biology and School of Informatics and Computing, Indiana University, Bloomington, IN 47405, USA; 6 Department of Integrative Biology, University of Texas, Austin, TX 78712, USA; 7 National Institute of Genetics, Mishima, Shizuoka, 411-8540, Japan; 8 Department of Genetics, Evolution and Environment, University College London, London, WC1E 6BT, UK; 9 Department of Molecular Biology and Ecology of Plants, Tel Aviv University, Tel-Aviv, 69978, Israel; 10 Department of Plant Biology, University of Illinois, Urbana, IL 61801, USA; 11 Department of Zoology, University of British Columbia, Vancouver, BC V6J 3S7, Canada; 12 Division of Human Biology, Fred Hutchinson Cancer Research Center, Seattle, WA 98109, USA; 13 Department of Biological Sciences, University of Idaho, Moscow, ID 83844, USA; 14 Department of Ecology and Evolution, University of Lausanne, CH-1015 Lausanne, Switzerland; 15 Institute of Evolutionary Biology, University of Edinburgh, Edinburgh, EH9 3JT, UK; 16 Department of Ecology, Evolution and Organismal Biology, Iowa State University, Ames, IA 50011, USA; 17 Department of Biological Sciences, University of Calgary, Calgary, AB T2N 1N4, USA

## Abstract

The vast majority of eukaryotic organisms reproduce sexually, yet the nature of the sexual system and the mechanism of sex determination often vary remarkably, even among closely related species. Some species of animals and plants change sex across their lifespan, some contain hermaphrodites as well as males and females, some determine sex with highly differentiated chromosomes, while others determine sex according to their environment. Testing evolutionary hypotheses regarding the causes and consequences of this diversity requires interspecific data placed in a phylogenetic context. Such comparative studies have been hampered by the lack of accessible data listing sexual systems and sex determination mechanisms across the eukaryotic tree of life. Here, we describe a database developed to facilitate access to sexual system and sex chromosome information, with data on sexual systems from 11,038 plant, 705 fish, 173 amphibian, 593 non-avian reptilian, 195 avian, 479 mammalian, and 11,556 invertebrate species.

## Background & Summary

Sexual reproduction is a nearly universal feature of eukaryotes, yet a remarkable diversity of sexual systems and sex determining (SD) mechanisms exists. The sexual system of a lineage has important evolutionary and ecological implications, affecting the levels of genetic variation maintained, the degree of inbreeding, the rate of adaptation to novel environments, as well as having longer-term consequences for the formation of new species and the risk of extinction. However, we know little about why and how different sexual systems have evolved. In order to remedy this knowledge gap, the Tree of Sex consortium, a working group of the National Evolutionary Synthesis Center (NESCent), has compiled existing information on sexual systems and sex determination mechanisms, focusing particularly on groups of plants and animals exhibiting variation. In addition to sexual system and mechanism of sex determination (see Tables 1 and 2 for ontology), traits were collated to allow researchers to correlate transitions in mating systems to features of the genome (e.g., chromosome number, ploidy level) and life history (e.g., growth form, life form). To maximize ease of re-use, the data have been deposited in a public repository (see Data Records) and in a trait database custom built by NESCent (TraitDB; http://purl.org/nescent/treeofsex) to which additional data may be uploaded. These data are suitable for in depth comparative analyses of the factors influencing the evolution of sexual systems as well as analyses of the impact of sexual system on, e.g., species ranges, invasiveness, or extinction risk.

To build the database, we surveyed the literature, on-line databases, and expert scientists to obtain species-level descriptions of the traits listed in Tables 1 and 2. For many species, data were obtained from the initial taxonomic description of the species or from subsequent primary literature about the species. While these data are readily available in hard copy in many libraries, it is prohibitively time consuming to track down information species-by-species for use in analyses across broad taxonomic scales. Furthermore, some data are not available in English or require expert interpretation, reducing the usability of the data. For example, botanical knowledge is needed to recognize that plants with ‘perfect flowers’ or ‘monoclinous’ plants are hermaphroditic, with male and female parts contained in the same flowers, or that trioecy and polygamodioecy can both be used to refer to plants with males, females, and hermaphrodites. The Tree of Sex consortium set out to build a database that would make the data accessible and downloadable, using a common ontology describing the traits of interest (Tables 1 and 2).

**Figure 1:**
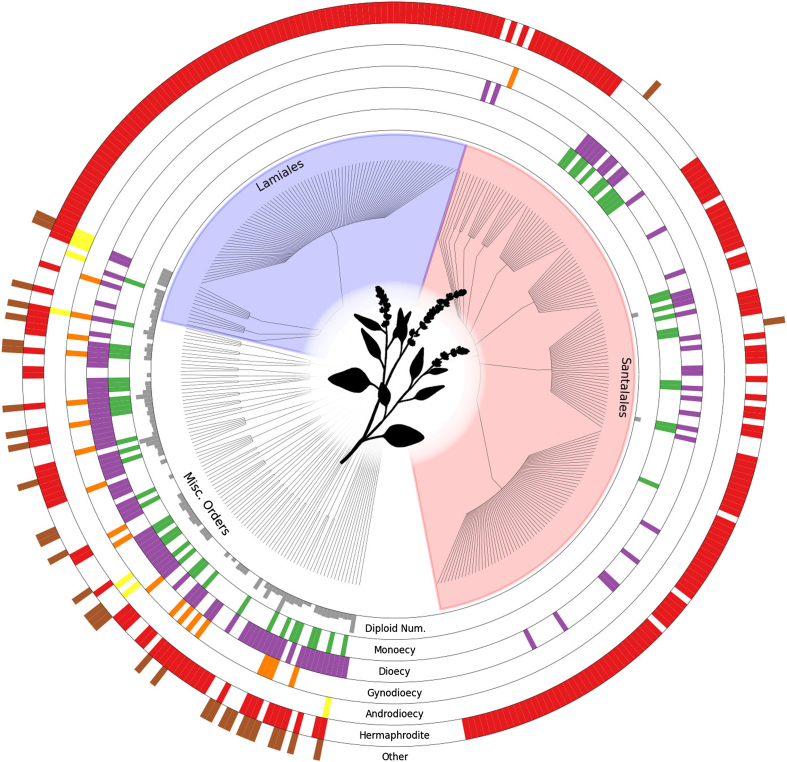
**Distribution and sample of plant data from the Tree of Sex Database**. Tree structure is derived from taxonomy, where each tip represents all species in a single genus. Diploid chromosome number is indicated by the height of the innermost ring; all other rings indicate the presence or absence of the trait named at the base of the ring. The ‘Other’ ring includes the states: apomictic, gynomonoecy, andromonoecy, polygamodioecy, and polygamomonoecy. The sexual trait data displayed in the rings is based on 11,038 plant entries.

**Figure 2:**
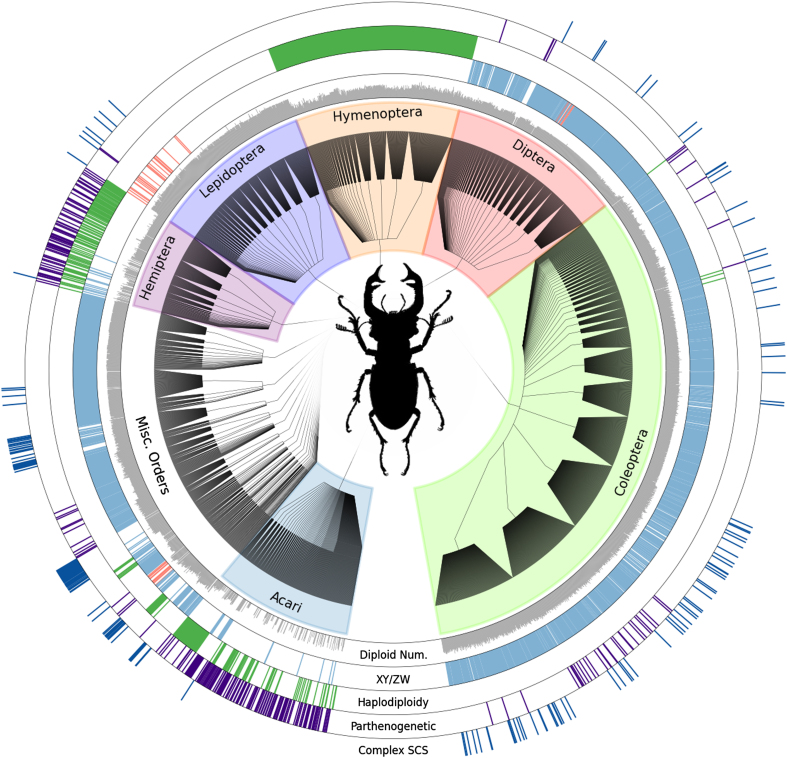
**Distribution and sample of invertebrate** **data from the Tree of Sex Database**. The XY/ZW ring is colored blue for XY and red for ZW taxa. Complex SCS indicates species with complex sex chromosome karyotypes (e.g., X_1_X_2_Y). The sexual trait data displayed in the rings is based on 11,556 invertebrate entries. Remaining features as in Figure 1.

**Figure 3:**
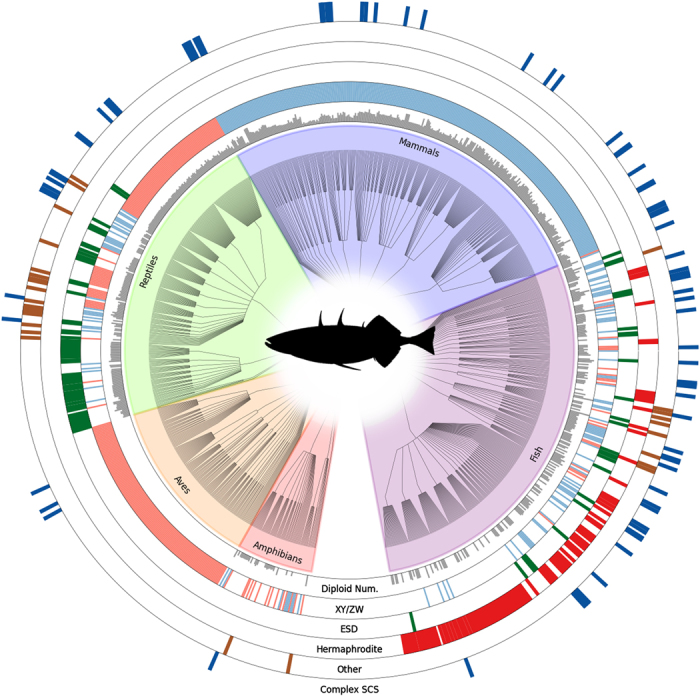
**Distribution and sample of vertebrate data from the Tree of Sex Database**. The ‘Other’ ring includes parthenogenesis, gynogenesis, and hybridogenesis. The XY/ZW ring is colored blue for XY and red for ZW taxa. The sexual trait data displayed in the rings is based on 2,145 vertebrate entries. Remaining features as in Figures 1 and 2.

For some taxa (Coleoptera, Hymenoptera, Diptera, Acari), we have endeavored to make our database comprehensive, representing nearly all known data about the sexual traits compiled. For plants, the database includes 382 genera (348 with data on sexual systems), although we concentrated our data collection on 77 clades (primarily genera), which were known to be variable for sexual system. For these 77 clades, the database has high coverage of the available information, enabling users to address questions about the impact of sexual system on evolutionary and ecological processes. Many genera of plants and invertebrates, however, remain poorly covered in the current database. For vertebrates, our coverage has focused on species with information about the mechanism of sex determination. Figures 1, 2 and 3 provide a summary of data currently available in the database. While the database is not complete, it provides a framework within which additional data can be added by the community. Such a collective effort is needed to fill in details about sexual systems across the Tree of Life.

The Tree of Sex database facilitates comparative analyses exploring hypotheses about the evolutionary factors driving transitions among sexual systems. Examples of the type of questions that are being addressed using the database are:

- *Do hermaphrodites diversify more rapidly than species with separate sexes (dioecy)?* Information in the database, coupled with phylogenetic information, is being used to determine the impact of dioecy on speciation and extinction rates across multiple genera of plants.
- *Does environmental sex determination place species at heightened risk of extinction, especially in the face of a changing climate?* The database allows us to measure the impact of environmental versus genetic sex determination on extinction risk in both turtles and squamates (lizards plus snakes) and to compare extinction risks among groups with different climatic histories.
- *Are some sexual systems more transient than others?* By mapping changes in sexual systems to the tree of life, we are assessing whether some transitions are more likely to happen than others (e.g., are ZW systems more likely to transition to XY than vice versa? are gynodioecious species more likely to transition to dioecy than the reverse?).
- *Does the mechanism of sex determination affect genomic evolution?* The data are being used to assess which taxa are most likely to undergo fusions between autosomes and sex chromosomes to test ideas about the drivers of fusion events.
- *What factors influence loss of Y chromosomes?* In some taxa, Y chromosomes are readily lost, while in others they persist; we have explored the tempo and mode of Y chromosome loss^1^.
- *Does sociality affect the evolution of chromosome number?* Eusocial lifestyles may create selection pressure for increased recombination and indirectly increase the number of chromosomes; we are performing a comparative analysis to test this hypothesis.

By synthesizing existing data on sexual systems, the database will allow biologists to identify the evolutionary and ecological processes that underlie the remarkable diversity in sexual systems across the tree of eukaryotic life. The database will also facilitate rapid identification of suitable taxonomic groups that contain variation in traits of interest for future studies of sexual systems and sex determination.

## Methods

Sexual system, karyotype, genome size, ploidy, and life history data were collated from taxonomic accounts, books on flora and fauna, online scientific databases, and the primary scientific literature (sources from the literature were preferred when available). For each species and trait, state values and data sources were entered in the database. In cases where within-species variation in a trait was documented, variants were included in the database, except where noted as ‘rare.’ A notes field in the database for each species allows additional information, such as taxonomic uncertainty or rare variants, to be listed. We avoided using generic information about higher-level taxa (e.g., ‘family X is dioecious’) to inform the trait states of a species, unless the source explicitly listed the species when describing the characteristics of the higher-level taxon.

### Vertebrate traits

Data for fish, non-avian reptiles, amphibians, mammals, and birds were compiled from literature searches. Birds appear to be uniform with respect to their sex determination system and were only included if they had specific karyotypic information to distinguish between ZW or complex ZW (e.g., Z1Z2W) systems. Sources included books with karyotype information^2–11,2–11,2–11,2–11,2–11,2–11,2–11,2–11,2–11,2–11^, online databases^12^, review papers^13,14^, and primary research papers. If different values were identified for a particular trait in a given species, multiple entries with the different values are provided for that species.

### Invertebrate traits

We performed an extensive literature search using Google Scholar and Web of Science and compiled ~12,000 entries across all orders of hexapods as well as mites. The invertebrate data are drawn from approximately 453 published records including primary research papers, review papers and previous compilations in books. We also incorporated additional data from existing databases (i.e., ScaleNet for scale insects^15^). For each group in our database we performed literature searches using order and family names in conjunction with the terms: karyotype, cytotaxonomy, cytogenetic, parthenogenesis, haplodiploidy, polyploidy, sex chromosomes and chromosome number. To the extent possible, we reconciled historical karyotype data with currently accepted taxonomy.

### Plant traits

The dataset focused on 77 angiosperm clades (primarily at the genus level) exhibiting inter-specific variation in sexual systems to allow species-level analysis of sexual diversity. Genera were chosen from the list containing species with separate sexes compiled by Renner and Ricklefs^16^, with additional genera from Miller and Venable^17^, plus genera known to have species with sex chromosomes^18^, and additional taxa known to the authors to be variable in sexual system. We focused on clades that (a) had at least 15 species, (b) were thought to have at least three dioecious species and three non-dioecious species according to Renner and Ricklefs^16^, and (c) have sufficient sequence information in GenBank for phylogenetic analyses based on NCBI taxonomy^19^. Species names were checked against The Plant List (Version 1.1)^20^. For each of these clades, we gathered data on sexual system, life history, growth form, woodiness and chromosome numbers from four main sources: (1) searching through monographs and local floras (either printed or accessed online via, e.g., eflora.org), (2) detailed search of the primary literature (454 papers), (3) additional online sources (e.g., PLANTS Database^21^), and (4) consulting with experts with knowledge of the group in question. Finally, we included the extensive information on reproductive traits in parasitic plants from Bellot and Renner^22^ and on woodiness from Zanne *et al.*^23,24^

## Data Records

### Data record 1

The database files (May 19, 2014 version) in csv format were uploaded to Dryad (Data Citation 1). Separate files are available for the vertebrate, invertebrate, and plant data. Additional notes about the data and pers. comm. files are in this Dryad record as a zip file. Taxonomic information is provided to ensure unique species identity of each record (Order, Family, Genus, species).

### Data record 2

The trait data were also uploaded to TraitDB^25^ within the Tree of Sex project (Data Citation 2). TraitDB is a searchable MySQL-based database, custom built by NESCent, which allows authorized administrators to upload additional data and delete incorrect records. Contributors of additional data should go to treeofsex.org for file configuration information.

## Technical Validation

Automated data entries were manually curated to ensure validity. Sub-samples of the records were checked by a different author from the data collector. For the invertebrate and plant data sets, custom scripts were written to identify potentially inconsistent entries (e.g., rows for species with XY karyotypes where the number of chromosomes should be even but was odd, rows specifying that the species is parthenogenetic but describing a male karyotype, species that were said to be both trees and herbs, etc.). All records that appeared to be inconsistent were checked against the original sources to confirm that the entries represent either true trait variation or variation in expert opinion. The on-line database may also be expanded and corrected, as new information becomes available.

## Usage Notes

The data are available for download as flat csv files from Dryad (May 19, 2014 version) and from TraitDB. These data may be imported into R^26^ for use in comparative analyses, e.g., using *ape*^27^, *diversitree*^28^, GraPhlAn^29^ (as used to generate Figures 1, 2 and 3), or other packages. The Tree of Sex Consortium places no restrictions on the re-use of the data; we request details of any publications that make substantial use of the database for posting on the website.

## Additional information

**How to cite this article:** The Tree of Sex Consortium. Tree of Sex: A database of sexual systems. *Sci. Data* 1:140015 doi: 10.1038/sdata.2014.15 (2014).

## Supplementary Material



## Figures and Tables

**Table 1:** Sexual system database ontology in plants.

**Trait**	**States**
Sexual system^a^	Hermaphrodite, monoecy, dioecy, gynodioecy, androdioecy, gynomonoecy, andromonoecy, polygamodioecy, polygamomonoecy, apomictic, other^b^
Genotypic (sex determination)	Male heterogametic, female heterogametic, GSD, polygenic
Karyotype	ZO, ZW, XY, XO, WO, homomorphic, complex XY (e.g., X1X2Y), complex ZW (e.g., Z1Z2W)
Molecular basis	Dosage, Y dominant, W dominant
Selfing	Self incompatible, self compatible
Growth form	Herb, shrub, tree, herbaceous vine, liana/woody vine
Woodiness^c^	W woody, H herbaceous, variable
Woodiness count^c^	#W; #H; #variable
Life form	Annual, perennial
Chromosome number^d^	(List of records for counts)
Chromosome number^d^ (minimum)	(integer number)
Chromosome number^d^ (mean)	(real number)

^a^Sexual system is the morphological system. In some species, hermaphrodites function primarily as males or primarily as females, but this information is not known for the majority of species in the database.

^b^Sexual system states include: Hermaphrodite, plants whose flowers have both male and female parts. Monoecy, plants have separate male and female flowers on the same plant. Dioecy, all plants are either female or male. Gynodioecy, both female and hermaphrodite plants present. Androdioecy, both male and hermaphrodite plants present. Gynomonoecy, female and hermaphrodite flowers within a plant. Andromonoecy, male and hermaphrodite flowers within a plant. Polygamodioecy, male, female, and hermaphrodite plants present. Polygamomonoecy, male, female, and hermaphrodite flowers within a plant. Apomictic, asexual/parthenogenetic.

^c^As reported by Zanne *et al.*^23,24^.

^d^Separate columns indicate gametophytic (after meiosis; ‘haploid’ number) and sporophytic chromosome counts (before meiosis; ‘diploid’ number).

**Table 2:** Sexual system database ontology in Animals.

**Trait**	**States**
Sexual system	(as in Table 1 a)
Genotypic (sex determination)	(as in Table 1)
Karyotype	(as in Table 1)
Molecular basis	(as in Table 1)
Chromosome number (female)	(integer number)
Chromosome number (male)	(integer number)
Predicted ploidy	1,2,3,4
Haplodiploidy (sex determination)	Arrhenotoky, paternal genome elimination, other
Environmental (sex determination)	TSD, TSD Ia, TSD Ib, TSD II, size, density, pH, ESD_other^b^
Polyfactorial (sex determination)	Yes, no

^a^In animals, gonochorous is used in place of dioecy.

^b^TSD: general term when reaction norm with temperature is not specified. TSD Ia: males produced at low temperatures and females at high temperature. TSD Ib: females produced at low temperatures and males at high temperature. TSD II: females produced at low and high temperatures, males produced at intermediate values.
